# Supplementary material for: Residential Greenspace Is Associated with Lower Levels of Depressive and Burnout Symptoms, and Higher Levels of Life Satisfaction: A Nationwide Population-Based Study in Sweden
Source: Int J Environ Res Public Health. 2022 May 6;19(9):5668. doi: 10.3390/ijerph19095668 (PMC9100967; doi:10.3390/ijerph19095668)
Supplement: Supplementary file 1 [file ijerph-19-05668-s001.zip › ijerph-1685835-supplementary.pdf]

Supplementary information

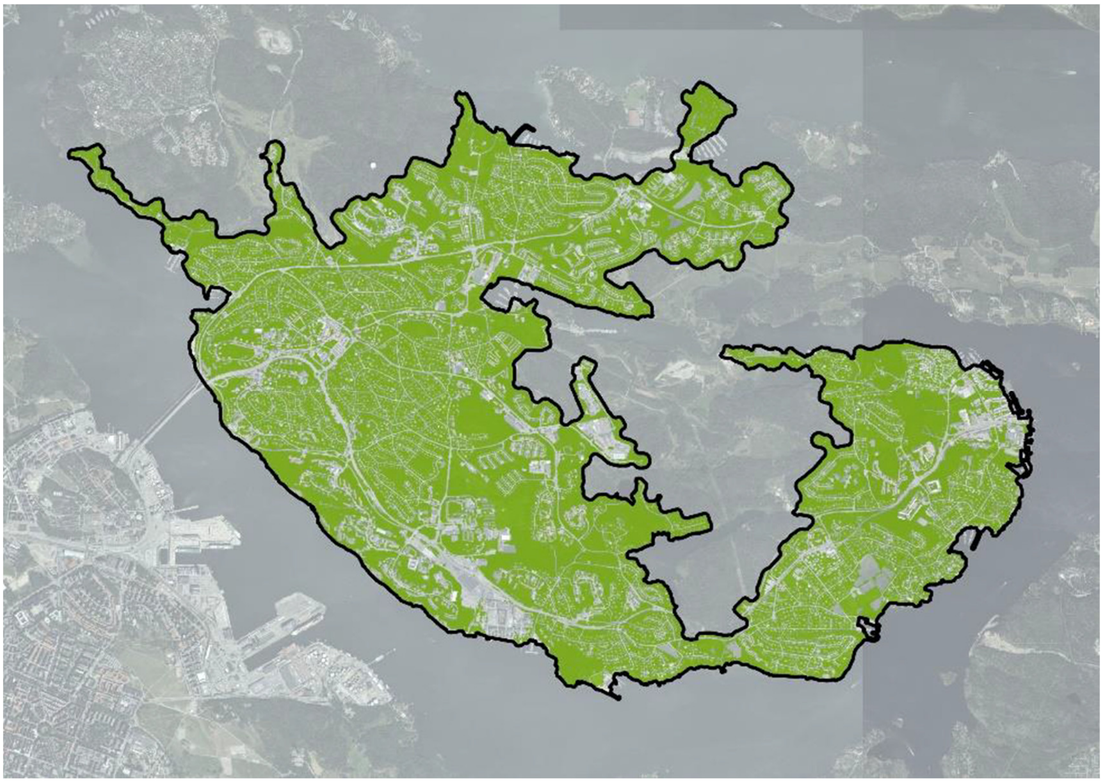

Supplementary Figure S1. An example map of total greenspace land cover (green), and built space land cover (in grey, incl. buildings, paved land, road and rail networks) in one of the greenest urban areas in Sweden, with ca. 70% total greenspace (the urban area of Lidingö municipality, a suburb of Stockholm City) [32]: Source: SCB, ortofoto © Lantmäteriet.

Table S1. Associations between residential greenspace at 50 m, 100 m 300 m and 500 m buffers, and depressive symptoms (subsample: working).

	**50 m Buffer**				**100 m Buffer**				**300 m Buffer**				**500 m Buffer**			
Model, N= 10 170	β	B	Lower, upper (95% CI of B)	p	β	B	Lower, upper (95% CI of B)	p	β	B	Lower, upper (95% CI of B)	p	β	B	Lower, upper (95% CI of B)	p
1	−0.015	−0.004	−0.009, 0.001	0.119	−0.003	−0.001	−0.006, 0.004	0.767	−0.008	−0.002	−0.008, 0.003	0.438	−0.009	−0.003	−0.009, 0.003	0.363
2	−0.008	−0.002	−0.007, 0.003	0.402	0.002	0.001	−0.005, 0.006	0.842	−0.006	−0.002	−0.007, 0.004	0.551	−0.008	−0.002	−0.008, 0.003	0.425
3	−0.009	−0.002	−0.007, 0.003	0.364	0.002	0.000	0.005, 0.006	0.867	−0.006	−0.002	−0.007, 0.004	0.549	−0.008	−0.002	−0.008, 0.003	0.411
4	−0.009	−0.002	−0.007, 0.003	0.360	0.002	0.000	−0.005, 0.006	0.877	−0.007	−0.002	-.0008, 0.004	0.502	−0.009	−0.003	−0.009, 0.003	0.353
5	0.007	0.002	−0.003, 0.007	0.471	0.018	0.005	0.000, 0.010	0.066	0.007	0.002	−0.004, 0.008	0.457	.003	.001	−0.005, 0.007	0.761

Note: Variables in Model 1= residential greenspace, unadjusted; Model 2= Model 1 + age, sex; Model 3= Model 2 + average neighborhood income; Model 4 = Model 3 + individual income; Model 5 = Model 4 + relationship status

B= unstandardized beta coefficient, β = standardized beta coefficient beta coefficient

Table S2. Associations between residential greenspace at 50 m, 100 m 300 m and 500 m buffers, and burnout symptoms (subsample: working).

	**50 m Buffer**	**100 m Buffer**	**300 m Buffer**	**500 m Buffer**

Model, N= 10 187	β	B	Lower, upper (95% CI of B)	p	β	B	Lower, upper (95% CI of B)	p	β	B	Lower, upper (95% CI of B)	p	β	B	Lower, upper (95% CI of B)	p
1	−0.011	−0.001	−0.002, 0.001	0.247	0.000	0.000	−0.001, 0.001	0.964	0.001	0.000	−0.001, 0.002	0.895	−0.001	0.000	−0.002, 0.001	0.950
2	−0.004	0.000	−0.001, 0.001	0.672	0.005	0.000	−0.001, 0.002	0.583	0.003	0.000	−0.001, 0.002	0.737	0.001	0.000	−0.001, 0.002	0.937
3	−0.006	0.000	−0.002, 0.001	0.524	0.004	0.000	−0.001, 0.002	0.650	0.003	0.000	−0.001, 0.002	0.742	0.000	0.000	−0.001, 0.001	0.994
4	−0.006	0.000	−0.002, 0.001	0.512	0.004	0.000	−0.001, 0.002	0.671	0.002	0.000	−0.001, 0.002	0.841	−0.002	0.000	−0.002, 0.001	0.857
5	0.007	0.000	−0.001, 0.002	0.467	0.018	0.001	0.000, 0.003	0.065	0.014	0.001	0.000, 0.002	0.161	0.008	0.001	−0.001, 0.002	0.389

Note: Variables in Model 1= residential greenspace, unadjusted; Model 2= Model 1 + age, sex; Model 3= Model 2 + average neighborhood income; Model 4 = Model 3 + individual income; Model 5 = Model 4 + relationship status

B= unstandardized beta coefficient, β = standardized beta coefficient beta coefficient

Table S3. Associations between residential green- and blue space at 50 m, 100 m 300 m and 500 m buffers, and depressive symptoms (whole sample/population).

	**50 m Buffer**				**100 m Buffer**				**300 m Buffer**				**500 m Buffer**			
Model, N= 14 290	β	B	Lower, upper (95%	p	β	B	Lower, upper (95%	p	β	B	Lower, upper (95%	p	β	B	Lower, upper (95%	p

			CI of B)				CI of B)				CI of B)				CI of B)	
1	−0.027	−0.007	−0.011, −0.003	0.001	−0.019	−0.005	−0.010, −0.001	0.023	−0.026	−0.008	−0.013, −0.003	0.002	−0.029	−0.009	−0.015, −0.004	0.001
2	−0.017	−0.004	−0.009, 0.000	0.038	−0.010	−0.003	−0.007, 0.002	0.215	−0.019	−0.006	−0.011, −0.001	0.019	−0.022	−0.007	−0.012, −0.002	0.007
3	−0.018	−0.005	−0.009, 0.000	0.028	−0.010	−0.003	−0.007, 0.002	0.216	−0.017	−0.005	−0.010, 0.000	0.036	−0.020	−0.006	−0.012, −0.001	0.017
4	−0.017	−0.005	−0.009, 0.000	0.034	−0.010	−0.003	−0.007, 0.002	0.222	−0.018	−0.006	−0.011, −0.001	0.028	−0.021	−0.007	−0.012, −0.002	0.011
5	−0.001	0.000	−0.004, 0.004	0.940	−0.008	0.002	−0.002, 0.007	0.360	−0.003	−0.001	−0.006, −0.004	0.714	−0.008	−0.003	−0.008, 0.003	0.349

Note: Variables in Model 1= residential green- and blue space, unadjusted; Model 2= Model 1 + age, sex; Model 3= Model 2 + average neighborhood income; Model 4 = Model 3 + individual income; Model 5 = Model 4 + relationship status

β= Standardized beta coefficient; B= Unstandardized beta coefficient

Table S4. Associations between residential green- and blue space at 50 m, 100 m 300 m and 500 m buffers, and depressive symptoms (subsample: non-working).

	**50 m Buffer**				**100 m Buffer**				**300 m Buffer**				**500 m Buffer**			
Model, N= 4 120	β	B	Lower, upper (95% CI of B)	p	β	B	Lower, upper (95% CI of B)	p	β	B	Lower, upper (95% CI of B)	p	β	B	Lower, upper (95% CI of B)	p

1	−0.059	−0.015	−0.023, −0.007	0.000	−0.056	−0.015	−0.024, −0.007	0.000	−0.058	−0.018	−0.027, −0.008	0.000	−0.065	−0.021	−0.031, −0.011	0.000
2	−0.040	−0.010	−0.018, −0.003	0.008	−0.038	−0.010	−0.018, −0.002	0.014	−0.042	−0.013	−0.022, −0.004	0.006	−0.050	−0.016	−0.025, −0.006	0.001
3	−0.042	−0.011	−0.018, −0.003	0.006	−0.037	−0.010	−0.018, −0.002	0.015	−0.037	−0.011	−0.020, −0.002	0.015	−0.044	−0.014	−0.024, −0.004	0.004
4	−0.043	−0.011	−0.019, −0.003	0.004	−0.039	−0.011	−0.019, −0.003	0.009	−0.041	−0.013	−0.022, −0.004	0.006	−0.048	−0.015	−0.025, −0.006	0.001
5	−0.025	−0.006	−0.014, 0.001	0.095	−0.021	−0.006	−0.014, 0.003	0.176	−0.025	−0.008	−0.017, 0.001	0.102	−0.034	−0.011	−0.020, −0.001	0.026

Note: Variables in Model 1= residential green- and blue space, unadjusted; Model 2= Model 1 + age, sex; Model 3= Model 2 + average neighborhood income; Model 4 = Model 3 + individual income; Model 5 = Model 4 + relationship status

β= Standardized beta coefficient; B= Unstandardized beta coefficient

Table S5. Associations between residential green- and blue space at 50 m, 100 m 300 m and 500 m buffers, and depressive symptoms (subsample: working).

	**50 m Buffer**				**100 m Buffer**				**300 m Buffer**				**500 m Buffer**			
Model, N= 10 170	β	B	Lower, upper (95% CI of B)	p	β	B	Lower, upper (95% CI of B)	p	β	B	Lower, upper (95% CI of B)	p	β	B	Lower, upper (95% CI of B)	p
1	−0.016	−0.004	−0.009, 0.001	0.108	−0.006	−0.002	−0.007, 0.004	0.563	−0.015	−0.005	−0.011, 0.001	0.138	−0.015	−0.005	−0.011, 0.001	0.124

2	−0.008	−0.002	−0.007, 0.003	0.412	0.000	0.000	−0.005, 0.005	0.969	−0.011	−0.003	−0.009, 0.003	0.281	−0.012	−0.004	−0.010, 0.002	0.225
3	−0.009	−0.002	−0.007, 0.003	0.377	0.000	0.000	−0.005, 0.005	0.973	−0.010	−0.003	−0.009, 0.003	0.330	−0.010	−0.003	−0.010, 0.003	0.286
4	−0.009	−0.002	−0.007, 0.003	0.371	0.000	0.000	−0.005, 0.005	0.978	−0.010	−0.003	−0.009, 0.003	0.302	−0.011	−0.004	−0.010, 0.003	0.245
5	0.008	0.002	−0.003, 0.007	0.449	0.017	0.005	−0.001, 0.010	0.082	0.004	0.001	−0.005, 0.007	0.676	0.001	0.000	−0.006, 0.007	0.924

Note: Variables in Model 1= residential green- and blue space, unadjusted; Model 2= Model 1 + age, sex; Model 3= Model 2 + average neighborhood income; Model 4 = Model 3 + individual income; Model 5 = Model 4 + relationship status

β= Standardized beta coefficient; B= Unstandardized beta coefficient

Table S6. Associations between residential green- and blue space at 50 m, 100 m 300 m and 500 m buffers, and burnout symptoms (whole sample/population).

	**50 m Buffer**				**100 m Buffer**				**300 m Buffer**				**500 m Buffer**			
Model, N= 14 303	β	B	Lower, upper (95% CI of B)	p	β	B	Lower, upper (95% CI of B)	p	β	B	Lower, upper (95% CI of B)	p	β	B	Lower, upper (95% CI of B)	p
1	−0.021	−0.001	−0.002, 0.000	0.011	−0.015	−0.001	−0.002, 0.000	0.069	−0.020	−0.002	−0.003, 0.000	0.017	−0.023	−0.002	−0.003, 0.001	0.006
2	−0.009	−0.001	−0.002, 0.000	0.025	−0.005	0.000	−0.001, 0.001	0.569	−0.011	−0.001	−0.002, 0.000	0.161	−0.015	−0.001	−0.002, 0.000	0.067

3	−0.011	−0.001	−0.002, 0.000	0.170	−0.005	0.000	−0.001, 0.001	0.569	−0.008	−0.001	−0.002, 0.001	0.319	−0.010	−0.001	−0.002, 0.000	0.202
4	−0.010	−0.001	−0.002, 0.000	0.200	−0.004	0.000	−0.001, 0.001	0.588	−0.009	−0.001	−0.002, 0.001	0.273	−0.012	−0.001	−0.002, 0.000	0.155
5	−0.002	0.000	−0.001, 0.001	0.794	−0.009	0.001	−0.001, 0.002	0.288	−0.002	0.000	−0.001, 0.001	0.777	−0.002	0.000	−0.001, 0.001	0.823

Note: Variables in Model 1= residential green- and blue space, unadjusted; Model 2= Model 1 + age, sex; Model 3= Model 2 + average neighborhood income; Model 4 = Model 3 + individual income; Model 5 = Model 4 + relationship status

β= Standardized beta coefficient; B= Unstandardized beta coefficient

Table S7. Associations between residential green- and blue space at 50 m, 100 m 300 m and 500 m buffers, and burnout symptoms (subsample: non-working).

	**50 m Buffer**				**100 m Buffer**				**300 m Buffer**				**500 m Buffer**			
Model, N= 4 116	β	B	Lower, upper (95% CI of B)	p	β	B	Lower, upper (95% CI of B)	p	β	B	Lower, upper (95% CI of B)	p	β	B	Lower, upper (95% CI of B)	p
1	−0.050	−0.003	−0.005, −0.001	0.001	−0.049	−0.003	−0.005, −0.001	0.002	−0.056	−0.004	−0.007, −0.002	0.000	−0.059	−0.005	−0.007, −0.002	0.000
2	−0.027	−0.002	−0.004, 0.000	0.073	−0.026	−0.002	−0.004, 0.000	0.086	−0.035	−0.003	−0.005, 0.000	0.020	−0.038	−0.003	−0.005, −0.001	0.010
3	−0.028	−0.002	−0.004, 0.000	0.058	−0.025	−0.002	−0.004, 0.000	0.088	−0.031	−0.002	−0.005, 0.000	0.037	−0.034	−0.003	−0.005, −0.001	0.024

4	−0.029	−0.002	−0.004, 0.000	0.053	−0.027	−0.002	−0.004, 0.000	0.070	−0.034	−0.003	−0.005, 0.000	0.022	−0.037	−0.003	−0.005, −0.001	0.013
5	−0.019	−0.001	−0.003, 0.001	0.212	−0.016	−0.001	−0.003, 0.001	0.275	−0.025	−0.002	−0.004, 0.000	0.097	−0.029	−0.002	−0.005, 0.000	0.054

Note: Variables in Model 1= residential green- and blue space, unadjusted; Model 2= Model 1 + age, sex; Model 3= Model 2 + average neighborhood income; Model 4 = Model 3 + individual income; Model 5 = Model 4 + relationship status

β= Standardized beta coefficient; B= Unstandardized beta coefficient

Table S8. Associations between residential green- and blue space at 50 m, 100 m 300 m and 500 m buffers, and burnout symptoms (subsample: working).

	**50 m Buffer**				**100 m Buffer**				**300 m Buffer**				**500 m Buffer**			
Model, N= 10 187	β	B	Lower, upper (95% CI of B)	p	β	B	Lower, upper (95% CI of B)	p	β	B	Lower, upper (95% CI of B)	p	β	B	Lower, upper (95% CI of B)	p
1	−0.012	−0.001	−0.002, 0.000	0.211	−0.003	0.000	−0.002, 0.001	0.729	−0.007	−0.001	−0.002, 0.001	0.482	−0.010	−0.001	−0.002, 0.001	0.334
2	−0.004	0.000	−0.002, 0.001	0.652	0.003	0.000	−0.001, 0.001	0.780	−0.003	0.000	−0.002, 0.001	0.774	−0.006	0.000	−0.002, 0.001	0.524
3	−0.006	0.000	−0.002, 0.001	0.521	0.003	0.000	−0.001, 0.001	0.793	0.000	0.000	−0.001, 0.001	0.976	−0.002	0.000	−0.001, 0.001	0.853
4	−0.006	0.000	−0.002, 0.001	0.507	0.002	0.000	−0.001, 0.001	0.807	−0.001	0.000	−0.002, 0.001	0.940	−0.003	0.000	−0.002, 0.001	0.724
5	0.007	0.000	−0.001, 0.002	0.466	0.017	0.001	0.000, 0.002	0.091	0.011	0.001	−0.001, 0.002	0.248	0.007	0.001	−0.001, 0.002	0.477

Note: Variables in Model 1= residential green- and blue space, unadjusted; Model 2= Model 1 + age, sex; Model 3= Model 2 + average neighborhood income; Model 4 = Model 3 + individual income; Model 5 = Model 4 + relationship status

β= Standardized beta coefficient; B= Unstandardized beta coefficient

Table S9. Associations between residential green- and blue space at 50 m, 100 m, 300 m and 500 m buffers, and life satisfaction (whole sample/population).

	**50 m Buffer**				**100 m Buffer**				**300 m Buffer**				**500 m Buffer**			
Model, N= 14 279	β	B	Lower, upper (95% CI of B)	p	β	B	Lower, upper (95% CI of B)	p	β	B	Lower, upper (95% CI of B)	p	β	B	Lower, upper (95% CI of B)	p
1	0.035	0.002	0.001, 0.003	0.000	0.031	0.002	0.001, 0.003	0.000	0.035	0.003	0.001, 0.004	0.000	0.030	0.002	0.001, 0.004	0.000
2	0.032	0.002	0.001, 0.003	0.000	0.028	0.002	0.001, 0.003	0.001	0.033	0.003	0.001, 0.004	0.000	0.028	0.002	0.001, 0.004	0.001
3	0.034	0.002	0.001, 0.003	0.000	0.028	0.002	0.001, 0.003	0.001	0.029	0.002	0.001, 0.003	0.001	0.022	0.002	0.000, 0.003	0.009
4	0.033	0.002	0.001, 0.003	0.000	0.028	0.002	0.001, 0.003	0.001	0.029	0.002	0.001, 0.003	0.000	0.023	0.002	0.001, 0.003	0.005
5	−0.001	0.000	−0.001, 0.001	0.890	−0.008	−0.001	−0.002, 0.001	0.348	−0.001	0.000	−0.001, 0.001	0.896	−0.004	0.000	−0.002, 0.001	0.660

Note: Variables in Model 1= residential green- and blue space, unadjusted; Model 2= Model 1 + age, sex; Model 3= Model 2 + average neighborhood income; Model 4 = Model 3 + individual income; Model 5 = Model 4 + relationship status

β= Standardized beta coefficient; B= Unstandardized beta coefficient

Table S10. Associations between residential green- and blue space at 50 m, 100 m, 300 m and 500 m buffers, and life satisfaction (subsample: non-working).

	50 m Buffer				100 m Buffer				300 m Buffer				500 m Buffer			
Model, N= 4 102	β	B	Lower, upper (95% CI of B)	p	β	B	Lower, upper (95% CI of B)	p	β	B	Lower, upper (95% CI of B)	p	β	B	Lower, upper (95% CI of B)	p
1	0.056	0.004	0.002, 0.006	0.000	0.051	0.004	0.001, 0.006	0.001	0.054	0.004	0.002, 0.007	0.001	0.054	0.004	0.002, 0.007	0.001
2	0.049	0.003	0.001, 0.005	0.002	0.044	0.003	0.001, 0.005	0.005	0.048	0.004	0.001, 0.006	0.002	0.048	0.004	0.001, 0.006	0.002
3	0.052	0.003	0.001, 0.005	0.001	0.044	0.003	0.001, 0.005	0.005	0.042	0.003	0.001, 0.006	0.007	0.041	0.003	0.001, 0.006	0.009
4	0.053	0.003	0.001, 0.005	0.001	0.046	0.003	0.001, 0.005	0.003	0.046	0.004	0.001, 0.006	0.003	0.045	0.004	0.001, 0.006	0.004
5	0.024	0.002	0.000, 0.003	0.119	0.015	0.001	−0.001, 0.003	0.321	0.019	0.001	−0.001, 0.004	0.211	0.021	0.002	−0.001, 0.004	0.160

Note: Variables in Model 1= residential green- and blue space, unadjusted; Model 2= Model 1 + age, sex; Model 3= Model 2 + average neighborhood income; Model 4 = Model 3 + individual income; Model 5 = Model 4 + relationship status

β= Standardized beta coefficient; B= Unstandardized beta coefficient

Table S11. Associations between residential green- and blue space at 50 m, 100 m, 300 m and 500 m buffers, and life satisfaction (subsample: working).

50 m Buffer	100 m Buffer	300 m Buffer	500 m Buffer

Model, N= 10 177	β	B	Lower, upper (95% CI of B)	p	β	B	Lower, upper (95% CI of B)	p	β	B	Lower, upper (95% CI of B)	p	β	B	Lower, upper (95% CI of B)	p
1	0.027	0.002	0.000, 0.003	0.007	0.023	0.002	0.000, 0.003	0.018	0.029	0.002	0.001, 0.004	0.004	0.021	0.002	0.000, 0.003	0.032
2	0.025	0.002	0.000, 0.003	0.012	0.022	0.001	0.000, 0.003	0.027	0.027	0.002	0.001, 0.004	0.006	0.020	0.002	0.000, 0.003	0.042
3	0.027	0.002	0.001, 0.003	0.006	0.022	0.002	0.000, 0.003	0.024	0.023	0.002	0.000, 0.003	0.018	0.014	0.001	0.000, 0.003	0.148
4	0.028	0.002	0.001, 0.003	0.005	0.022	0.001	0.000, 0.003	0.024	0.024	0.002	0.000, 0.003	0.014	0.016	0.001	0.000, 0.003	0.110
5	−0.009	−0.001	−0.002, 0.003	0.352	−0.016	−0.001	−0.002, 0.000	0.113	−0.008	−0.001	−0.002, 0.001	0.418	−0.012	−0.001	−0.002, 0.001	0.207

Note: Variables in Model 1= residential green- and blue space, unadjusted; Model 2= Model 1 + age, sex; Model 3= Model 2 + average neighborhood income; Model 4 = Model 3 + individual income; Model 5 = Model 4 + relationship status

β= Standardized beta coefficient; B= Unstandardized beta coefficient
